# One-Step Microwave-Assisted Hydrothermal Preparation of Zn-ZnO(Nw)-rGO Electrodes for Supercapacitor Applications

**DOI:** 10.3390/ma16134536

**Published:** 2023-06-23

**Authors:** Cornelia Bandas, Mircea Nicolaescu, Mina Ionela Popescu, Corina Orha, Simona Căprărescu, Carmen Lazau

**Affiliations:** 1 Condensed Matter Department, National Institute for Research and Development in Electrochemistry and Condensed Matter, Timisoara, 1 Plautius Andronescu Street, 300254 Timisoara, Romania; cornelia.bandas@gmail.com (C.B.); nicolaescu.mircea13@yahoo.com (M.N.); mina.popescu37@gmail.com (M.I.P.); orha.corina@gmail.com (C.O.); 2 Department of Materials and Manufacturing Engineering, Faculty of Mechanical Engineering, Politehnica University of Timisoara Mihai Viteazu 1, 300222 Timisoara, Romania; 3 Department of Applied Chemistry and Engineering of Inorganic Compounds and Environment, Politehnica University of Timisoara, Blv. Vasile Parvan 6, 300223 Timisoara, Romania; 4 Department of Inorganic Chemistry, Physical Chemistry and Electrochemistry, Faculty of Chemical Engineering and Biotechnologies, University “Politehnica” of Bucharest, Polizu Street No. 1–7, 011061 Bucharest, Romania

**Keywords:** supercapacitor, ZnO nanowires, ZnO-rGO films, microwave-assisted hydrothermal

## Abstract

Zn-ZnO(Nw)-rGO hybrid electrodes for supercapacitor applications were successfully prepared in situ by a one-step microwave-assisted hydrothermal method by deposition of reduced graphene oxide (rGO) on the structure of ZnO nanowires grown on the Zn foil. During the hydrothermal treatment, two processes occur the reduction of graphene oxide (GO) and the deposition of rGO on the Zn-ZnO(Nw) support. The growth of ZnO nanowires was achieved by thermal oxidation below the melting point of the Zn foil in a controlled atmosphere. The as-obtained electrodes were assessed for structural, optical, and morphological properties by X-ray diffraction, Raman spectroscopy, ultraviolet-visible spectroscopy, SEM microscopy, and EDX analysis. The supercapacitor properties of the Zn-ZnO(Nw)-rGO hybrid electrodes were investigated by cyclic voltammetry, electrochemical impedance spectroscopy, and galvanostatic charge-discharge analysis. The CV curve reveals that the Zn-ZnO(Nw)-rGO hybrid structures work as negative electrodes and exhibit a non-ideal rectangle-like shape, suggesting that the as-synthesized structure behaves as a pseudo-capacitor. A maximum capacitance was determined to be 395.79 mF cm^−2^ at a scan rate of 5 mV s^−1^. Based on GCD analysis, the maximum specific capacitance of 145.59 mF cm^−2^ was achieved at a low power density of 2 mA cm^−2^. The cycle life assessment of the Zn-ZnO(Nw)-rGO hybrid electrode over a 250-cycle number was performed by CV and GCD analysis. The maximum retention rate of 120.86% was achieved from GCD analysis over 250 cycles for the Zn-ZnO(Nw)-rGO hybrid electrode.

## 1. Introduction

Presently, supercapacitors have been receiving considerable attention due to their fast charge-discharge rates and longer life cycle. Moreover, supercapacitors can provide a higher power density with a shorter charging time than batteries and a higher energy density than conventional dielectric capacitors [1]. Because of these properties, supercapacitors have been considered attractive energy storage devices and power providers for electronic devices and are featured in many applications, such as hybrid electric vehicles and electronic devices. Due to their high surface area and good conductivity, porous carbonaceous materials (graphite, activated carbon, and carbon nanotubes) are the most commonly used as electrode materials for supercapacitors. Among these carbon materials, graphene with two-dimensional nanosheets has attracted a lot of attention because of its large specific area, good mechanical strength, and thermal conductivity. It also exhibits potential applications in supercapacitors and other electrochemical energy storage devices [2]. Moreover, porous reduced graphene oxide (rGO) is perhaps the best choice as an electrode material for a supercapacitor because of its essential properties (highly exposed surface areas, high electrical conductivity, and excellent chemical stability) [3].

Metal oxide nanoparticles (MO), such as TiO_2_, SnO_2_, WO_3_, In_2_O_3_, ZnO, V_2_O_5_, NiO, and carbon-based materials, especially graphene, are very popular research subjects for supercapacitor electrode materials [4]. If MO can be combined with high-conductivity carbon materials, the critical problem of the low energy density of micro-supercapacitors (MSCs) can be solved. In literature data, the most common way to prepare MO NPs/graphene (MO/G) composites is by wet chemical methods, such as hydrothermal [5,6], solvothermal [7,8], sol-gel [9], and freeze-drying [10], because these technologies have strong operability and high product yields [11]. Lianbo Ma et al. investigated as supercapacitors a ternary nanocomposite based on Ag/MnO_2_/rGO that was synthesized by in situ growth of MnO_2_ nanoparticles on graphene oxide (GO) sheets, followed by co-reduction of Ag+ and GO [12]. Vanitha et al. synthesized a novel ternary Ag-decorated CeO_2_/rGO nanocomposite for the supercapacitor application via a facile hydrothermal method with polyvinylpyrrolidone (PVP) as a surface-directing agent. Due to the synergistic effect of these hybrid materials, their application both as photocatalysts and as supercapacitor materials for energy storage was increasingly promoted [13]. Zhang et al. have developed a supercapacitor based on reduced graphene oxide/Pt films through γ-ray irradiation that exhibited high specific capacitance, long cycle life, and high-rate capability [14]. ZnO is a suitable choice as a potential candidate for supercapacitors because it has a low cost, is environmentally friendly, and possesses a high exciton binding energy (60 meV) at room temperature and a wide direct band gap (3.37 eV); it typically crystallizes in hexagonal structures that enable a variety of uses in electrochemical applications [15,16], magnetic compounds [17], solar cells [18], photocatalysts [19], and gas sensing devices [20,21]. Further, the synergistic effect of the reduced GO (rGO) and MO materials exhibited superior electrochemical performance due to the combination of an electric double layer capacitor (EDLC) and pseudo capacitance behavior. In order to overcome the issues mentioned above, composite-based ZnO-rGO could improve the overall conductivity of the composite, electrochemical stability, and specific capacitance of ZnO [22]. The poor electrical conductivity of ZnO leads to the limitation of high-power performance capacity and its application in an energy storage system; instead, the synergism between graphene oxide and ZnO leads to obtaining a hybrid material that offers a powerful way to obtain a high specific capacity [23]. Jung et al. developed a simple and reliable laser-induced ZnO nanorod (NR)/reduced graphene oxide (rGO) technique for fabricating solid-state planar MSCs that exhibit both electric double-layer capacitance and pseudo-capacitance [4]. Buldu-Akturk et al. achieved good capacitive performance for the development of supercapacitor devices based on rGO/ZnO nanocomposites synthesized through high-energy ball milling, modified Hummers’ method, and demonstrated that rGO/ZnO nanocomposite could be a promising material for supercapacitor devices [24]. Jian et al. provide a new concept of introducing quantum dots into lithium-sulfur cathodes to achieve better electrochemical performance by successfully obtaining ZnO quantum dot-modified reduced graphene oxide (rGO@ZnO QDs) [25].

Several studies have reported different methods (hydrothermal, chemical solution synthesis, sonication method, or chemical vapor deposition) [26,27,28] to obtain composite materials based on ZnO-rGO from nanoscale to micrometer scale. In terms of controlled morphology and size, the microwave-assisted hydrothermal method is one of the most economical and efficient ways to synthesize hybrid composites, offering uniform heating, a high reaction rate, fast nucleation, and crystal growth, and the reduction of graphene oxide without using reducing agents. Aura S. Merlano et al. synthesized rGO / ZnO composites through the microwave-assisted hydrothermal method using different microwave irradiation times and different concentrations of Zn precursors [29]. Ting Lu et al. obtained the graphene-ZnO nanocomposite by microwave-assisted reduction of zinc ions in an aqueous solution with GO dispersion, and the results of electrochemical experiments indicated that the graphene-ZnO nanocomposite exhibits better capacitive performance compared to pure GO [30]. Ruiqi Gang et al. developed an easy one-step microwave-assisted gram-scale synthesis strategy to obtain 2D/2D ZnO/rGO hybrid photocatalysts that enhanced the photocatalytic activity of tetracycline photodegradation under ultraviolet light irradiation [31]. Other structures have been developed using the microwave-assisted hydrothermal method; namely, *C.* Lazau et al. successfully deposited rGO on titanium foil, obtaining Ti-TiO_2_-rGO composite structures suitable for electrochemical application [32]. The progress and novelty of this work consisted of the development of new hybrid electrodes based on metallic zinc decorated with nanowires of crystalline zinc oxide functionalized with reduced graphene oxide, directly in situ by microwave-assisted hydrothermal method. Because of the high surface-to-volume ratio of the as-synthesized Zn-ZnO(Nw)-rGO hybrid electrodes, the microwave-assisted hydrothermal method proves to be a promising technique for producing electrode materials suitable for supercapacitor applications. In summary, our research reports the viability of these Zn-ZnO(Nw)-rGO hybrid electrodes as counter-electrode materials for supercapacitor applications.

## 2. Materials and Methods

### 2.1. Chemicals

Graphene oxide (4 mg/mL) dispersed in H_2_O, zinc foil (thickness 0.25mm, 99.9% purity), acetone, and ethyl alcohol were purchased from Sigma-Aldrich Company (St. Louis, MO, USA). All the chemicals were of analytical grade and used as received.

### 2.2. Development of Hybrid Electrodes

**Figure 1:**
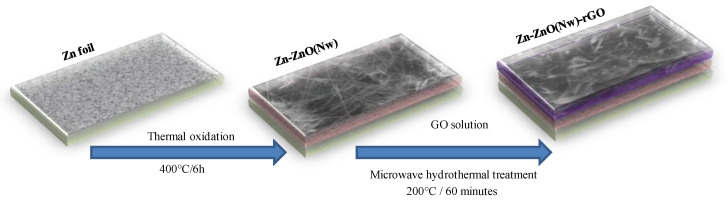
Schematic representation for the development of hybrid electrodes.

The Zn-ZnO(Nw)-rGO hybrid electrodes were developed by microwave-assisted hydrothermal reaction, which presumes two main processes: in situ reduction of GO to form rGO and in situ deposition of rGO films on metallic Zn-ZnO(Nw) surfaces. Figure 1 illustrates the schematic representation for the development of hybrid electrodes. The synthesis method of the Zn-ZnO(Nw) supports used in the experiments was reported in our previous work [33]. Within this research, working parameters were selected for Zn foil treatment: a temperature of 400 °C for 6 h in a controlled atmosphere oven (Ar and O_2_) with a gas flow rate of 100 mL / min. The microwave-assisted hydrothermal method consisted of an ethanolic solution of graphene oxide (1:1) stirred and ultrasounded for 30 min to obtain a homogeneous mixture, which was subsequently placed in a quartz autoclave together with Zn-ZnO(Nw) supports (effective area 1 × 1 cm^2^) with a degree of fullness of 10%. The autoclave was placed in a microwave oven at a heating temperature of 200 °C for 60 min (with a 20 min gradient temperature increase), with the oven power set to 1200 W (Anton Paar, Multiwave 3000 Microwave Digestion Oven, Austria). This process has the advantages of uniform deposition, good adhesion of rGO to the Zn-ZnO(Nw) crystalline layer, and preventing the exfoliation of rGO from the oxide layer. Finally, the as-obtained Zn-ZnO(Nw)-rGO hybrid electrodes were dried at 60 °C for 4 h.

### 2.3. Hybrid Electrode Characterization

X-ray diffraction analysis (XRD, PANalytical X’Pert PRO MPD Diffractometer, The Netherlands) with Cu-Kα radiation in the range *2theta* = 20–80° was used to investigate the crystalline structure of the hybrid structures. A UV-VIS spectrophotometer (PerkinElmer Lambda 950 UV/Vis, Shelton, WA, USA) with an integrating sphere in the range of 300–800 nm was used to record the optical characteristics. In order to identify the vibrational states of the as-synthesized structures, Raman spectroscopy was used with a Nanonics Imaging (Israel)—MultiProbe Imaging—MultiView 1000™ Platform (SPM) equipped with a 532 nm laser. The morphological and elemental properties of the structures were investigated by Scanning Electron Microscopy (SEM) using an FEI Inspect S model, The Netherlands coupled with an energy dispersive X-Ray analysis detector (EDX), both for Zn-ZnO(Nw) supports and Zn-ZnO(Nw)-rGO hybrid electrodes. The electrochemical characteristics were measured with a potentiostat/galvanostat, PGSTAT 302, Metrohm Autolab B.V. The Netherlands controlled with GPES 4.9 software using a classical three-electrode cell system in a 1M KOH solution, consisting of hybrid electrode Zn-ZnO(Nw)-rGO (an effective area of 1 cm^2^) as the working electrode, Ag/AgCl as the reference electrode, and platinum counter electrode.

## 3. Results and Discussion

**Figure 2:**
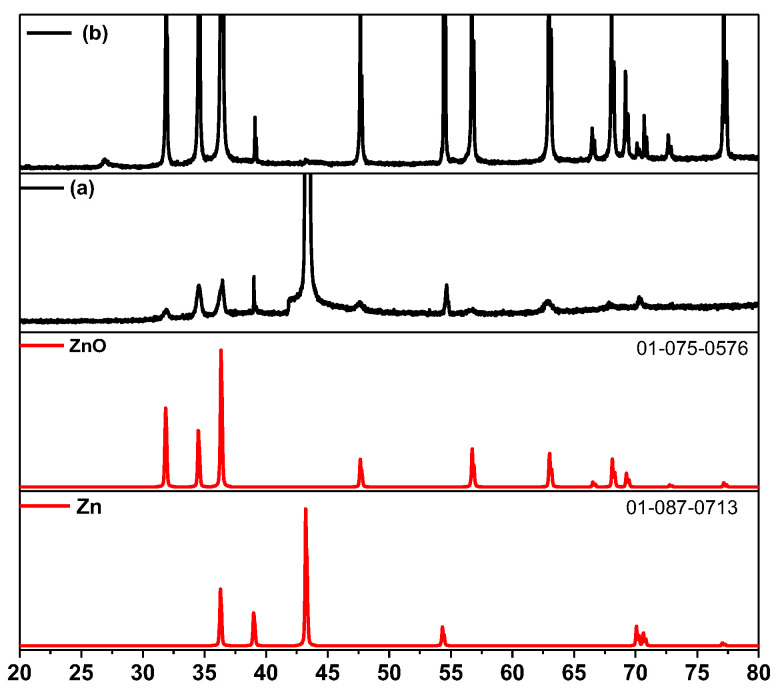
X-ray patterns for the (**a**) Zn-ZnO(Nw) supports and (**b**) Zn-ZnO(Nw)-rGO hybrid structures.

The X-ray patterns for the Zn-ZnO(Nw) structures and Zn-ZnO(Nw)-rGO hybrid electrodes are presented in Figure 2. Therefore, all peaks observed for the Zn-ZnO(Nw) support presented at 2theta: 31.96°, 34.54°, 36.43°, 47.65°, 56.76°, 63.04°, and 68.13° (JCPDS 01-075-0576), respectively, confirmed the hexagonal structure of the ZnO crystal. Moreover, specific peaks of Zn from the support foil were identified at 2theta: 39.0°, 43.25°, 54.40°, and 70.10° (JCPDS 01-087-0713). The presence of rGO in the Zn-ZnO(Nw)-rGO hybrid structures is confirmed by the slight peak at 2theta = 26.96°, proving the reduction of graphene oxide.

**Figure 3:**
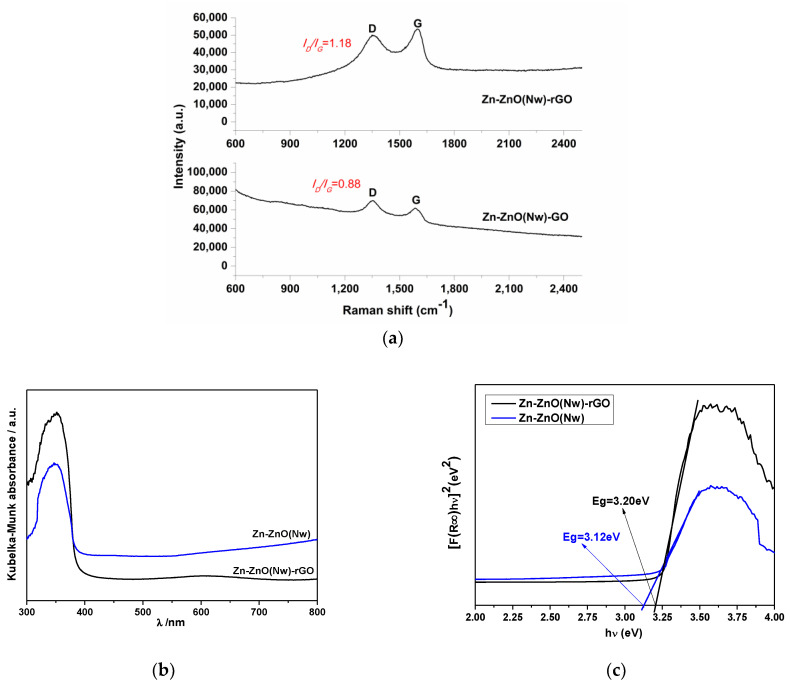
Raman spectra of the as-synthesized hybrid structure (**a**). *Kubelka-Munk* absorption for the as-synthesized structure (**b**). Tauc’s plot for *Eg* calculation for structure (**c**).

As shown in Figure 3a, Raman spectroscopy was also performed on Zn-ZnO(Nw)-GO and Zn-ZnO(Nw)-rGO hybrid structures to evaluate the vibrational states of reduced graphene oxide at wavenumbers specific to graphitized structures, i.e., from 600 to 2500 cm^−1^. Usually, there are two specific peaks specific to GO: one about 1300 cm^−1^ attributed to the D band arises from the defects present in the hexagonal structure, and another around 1500 cm^−1^ attributed to the G band corresponds to the *sp*^2^ hybridized carbon-carbon bonds. In the Raman spectra of Zn-ZnO(Nw)-GO, *I_D_*/*I_G_* was about 0.88, while in Zn-ZnO(N_W_)-rGO, the ratio was increasing to 1.18. The increase indicates the appearance of defects after the reduction process and the successful removal of functional oxygen groups [34], with the specific peak attributed to the reduced graphene oxide also confirmed by the XRD spectra (Figure 2). The absorption spectra of the Zn-ZnO(Nw)-GO and Zn-ZnO(Nw)-rGO structures are reported in Figure 3b, showing optical band-gap absorption, which can be attributed to intrinsic band-gap absorption of ZnO [35]. The *Eg* optical bandgap energy derived from the intersection of the straight line with the hν-axis of the Tauc plot was calculated in Figure 3c. Compared to the band-gap value of Zn-ZnO(Nw) supporting about *Eg* = 3.10 eV, in the case of Zn-ZnO(N_W_)-rGO, the value slightly increased due to the presence of rGO, about *Eg* = 3.20 eV. Furthermore, with the addition of rGO in the Zn-ZnO(Nw) matrix, the hybrid structure exhibits a more intense absorbance than only Zn-ZnO(Nw) supports [36].

**Figure 4:**
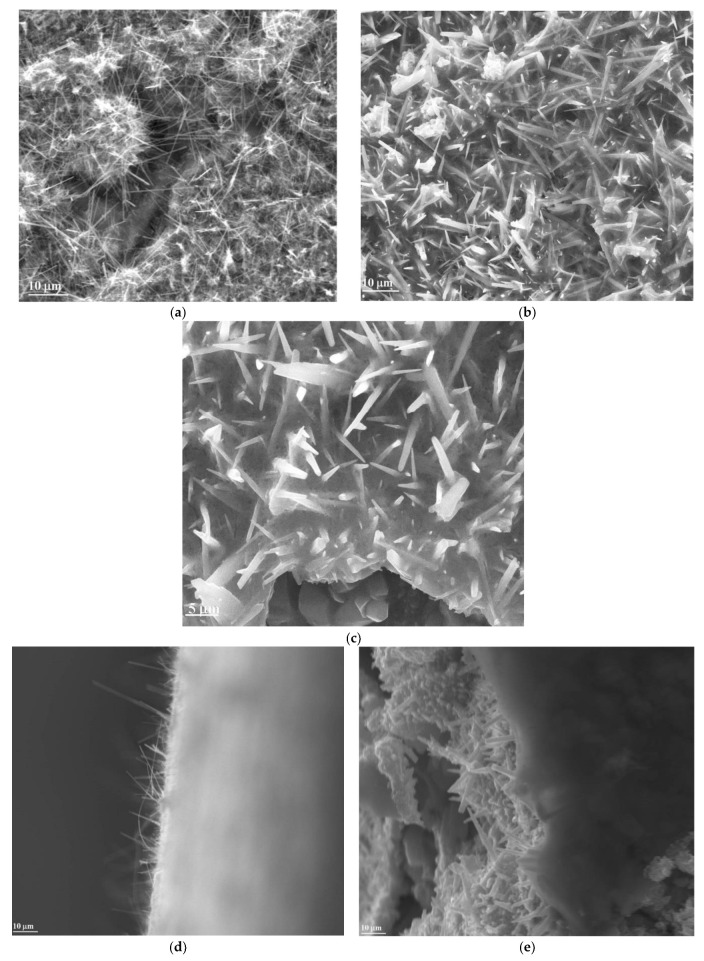

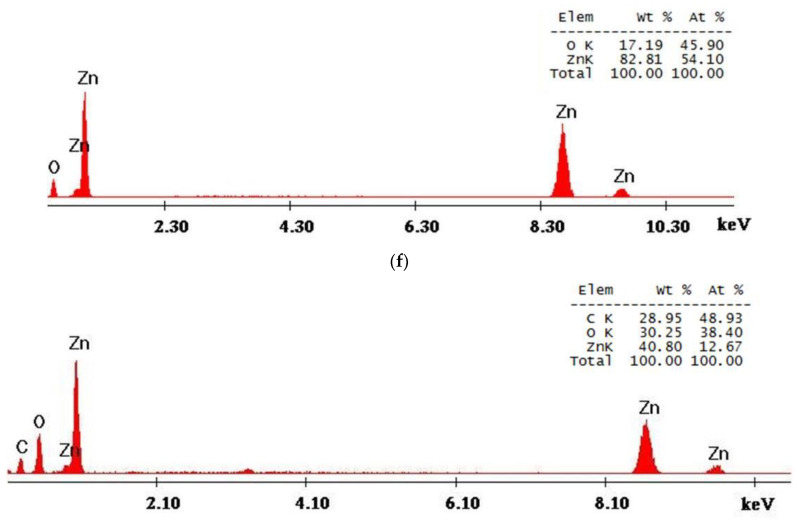
SEM morphologies of the Zn-ZnO(Nw) supports at a magnification of 6000× (**a**); SEM morphologies of the Zn-ZnO(Nw)-rGO hybrid structures at a magnification of 6000× (**b**) and 12,000× (**c**); Cross-sectional image for Zn-ZnO (Nw) support (**d**); Cross-sectional image for Zn-ZnO (Nw)-rGO hybrid electrode (**e**); EDX elemental analysis for Zn-ZnO(Nw) supports (**f**); and Zn-ZnO(Nw)-rGO hybrid structures (**g**).

From the SEM morphology (Figure 4a) of the Zn-ZnO support, it can be clearly seen that a high density of ZnO nanowires grew over the entire surface of the Zn foil with a random orientation. Moreover, the medium width of ZnO nanowires was measured from SEM images using imageJ Software (Version 1.53t). The as-measured values were 0.38 µm for the Zn-ZnO(Nw) support and 0.81 µm for the Zn-ZnO(Nw)-rGO hybrid electrode. After hydrothermal treatment in a microwave reaction, it was observed that ZnO nanowires were more dispersed and their width increased. The presence of reduced graphene oxide is evidenced by the transparent and thin layer that is not uniformly deposited over the Zn-ZnO(Nw) support (Figure 4b,c). Additionally, the length of the ZnO wires by conducting cross-sectional analysis was evaluated (Figure 4d,e). It was demonstrated that the medium length of ZnO nanowires from the Zn-ZnO(Nw) support was about 1.75 µm, and for the Zn-ZnO(Nw) hybrid electrode, it was about 1.61 µm, with no major difference being observed. In summary, it can be seen that rGO has been anchored to the surface of the Zn-ZnO(Nw) substrate, illustrating good contact between these two components of the hybrid structures. EDX elemental analysis (Figure 4f,g) confirms the presence of the chemical elements Zn and O on the Zn-ZnO support and Zn, O, and C from the Zn-ZnO(Nw)-rGO hybrid structure, respectively.

**Figure 5:**
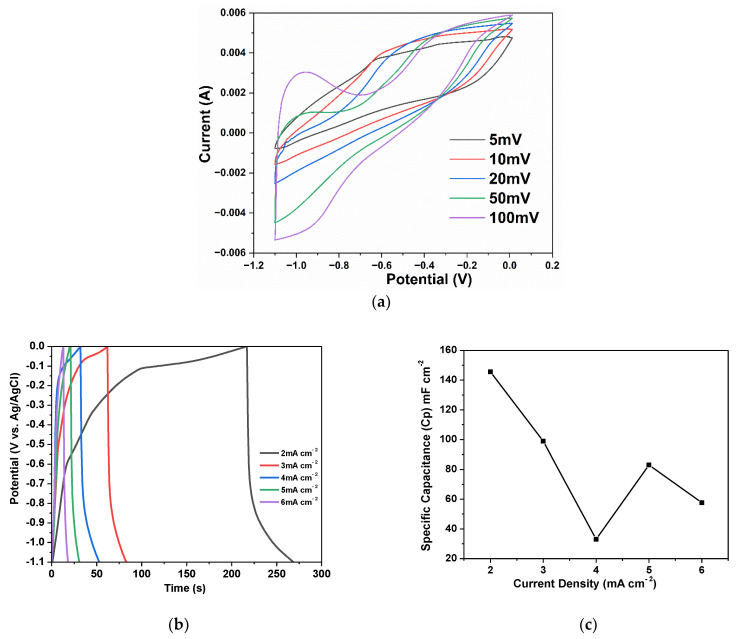
Cyclic voltammograms of the Zn-ZnO(Nw)-rGO electrode (**a**); GCD curves (**b**); and specific capacitance (**c**) of the Zn-ZnO(Nw)-rGO hybrid electrode at different current densities.

The electrochemical behavior of the Zn-ZnO(Nw)-rGO hybrid electrode was determined using cyclic voltammetry (CV), as shown in Figure 5a. A potential window range of −1.1 to 0 V was chosen for electrochemical measurements at a scan rate of 5, 10, 20, 50, and 100 mV s^−1^, the results indicating negative electrode behavior [37]. Experimental results obtained at different scan rates can provide valuable insights into the mechanism of charge storage and the performance of an electrode, usually from the shape of the cyclic voltammetry profile. In the case of the Zn-ZnO(Nw)-rGO negative electrode, the curves exhibited a non-ideal rectangular shape, indicating the presence of a pseudocapacitance effect in the electrode material [38]. Furthermore, as the scan rate increased, the current responses also changed, and the effects became more pronounced at higher scan rates. This behavior is confirmed by the curves presented in Figure 5a, where a pair of suspected redox peaks around -1V were observed to be more pronounced with the increasing scan rate [39]. It is clear that the relationship between the current and potential responses was non-linear, which strongly suggests the presence of faradaic pseudo-capacitance in the supercapacitor [40,41,42]. Based on equation 1, the highest capacitance from the CV study was determined to be 395.79 mF cm^−2^ at a scan rate of 5 mV s^−1^ [43].

(1) $$C_{p}=\frac{A}{kS\Delta V}$$

where *C*_P_ is the capacitance, *A* is the area under the curve, *k* is the scan rate, *S* represents the area of the active material (in cm^2^), and Δ*V* is the potential window.

The performance of the negative electrode presented in Figure 5b shows the galvanostatic charge-discharge (GCD) curves depending on time for different current densities (2, 3, 4, 5, and 6 mA cm^−2^). As presented in Figure 5a, it is obvious that an increase in current density results in a reduction in discharging time for the negative electrodes, which is in accordance with the behavior observed in other electrode materials for supercapacitor applications [44,45]. The non-uniform behavior observed throughout the charging/discharging process from the GCD profiles of the negative electrode is indicated by the pseudocapacitive behavior, consistent with the results obtained from the CV measurements of the as-produced Zn-ZnO(Nw)-rGO hybrid electrode [40,42].

To evaluate the pseudocapacitive behavior of the Zn-ZnO(Nw)-rGO electrode, the discharge region is commonly used to obtain the most accurate data. In this case, the discharge region of the GCD curve is composed of two main domains: a rapid voltage drop domain (due to the internal resistance) and an exponentially decreasing domain attributed to the pseudocapacitive behavior resulting from redox reactions at the interface between electrodes and electrolyte [42,45,46]. In Figure 5b, the specific capacitance (CSP) of the negative electrode, calculated from the GCD analysis, is plotted against the current density, following Equation (2) [47,48]:

(2) $$C_{SP}=\frac{I\Delta t}{\Delta VS}$$

where *I* represents the applied current (in mA), while Δ*t* and Δ*V* represent the discharging time (in s) and discharge voltage (in V), respectively. Additionally, *S* represents the area of the active material (in cm^2^).

The capacitance of the tested hybrid electrodes exhibits a dependence on the power densities; specifically, at 2 mA cm^−2^ and 4 mA cm^−2^, the specific capacitance of the Zn-ZnO(Nw)-rGO electrode decreases from 145.59 mF cm^−2^ to 32.97 mF cm^−2^ [48]. Furthermore, at power densities of 5 mA cm^−2^ and 6 mA cm^−2^, the specific capacitance of the Zn-ZnO(Nw)-rGO electrode initially shows a slight increase to 85.04 mF cm^−2^, followed by a decrease to 57.59 mF cm^−2^. The nonlinear variation in storage capacity depending on the current density can be attributed to the GCD analysis mechanism. At lower current densities, the plot exhibits higher pseudocapacitive behavior, where the electrochemical redox reaction at the interface between the electrode and electrolyte has a greater impact. However, with a slight increase in current density, the plot takes on a more triangular shape, so the redox reaction plays a smaller role in the overall interface mechanism [40]. This aspect suggests the presence of a slow and irreversible faradic reaction during the charge-discharge process. At high current density, the slow and irreversible faradaic reaction cannot follow the fast charge-discharge process, resulting in a slight increase in the specific capacitance [49].

**Figure 6:**
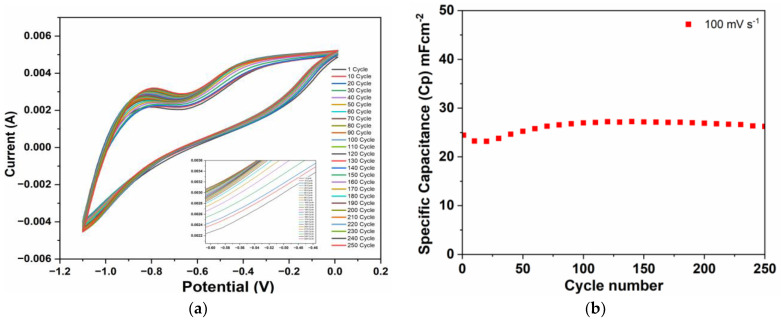
CV curves at 250 cycles of the Zn-ZnO(Nw)-rGO hybrid electrode at 100 mVs^−1^ scan rate (**a**) and calculated specific capacitance reported to cycle number (**b**).

The electrochemical stability of the Zn-ZnO(Nw)-rGO hybrid electrode was assessed by cyclic voltammetry (CV) measurements conducted over 250 cycles at a scan rate of 100 mV s^−1^, as presented in Figure 6a.

The Zn-ZnO(Nw)-rGO hybrid electrode showed a stable shape throughout the cycles, with a slight increase in the voltammetric area as the cycle number increased, suggesting that the as-tested electrode exhibits good cyclic stability under a constant scan rate. For a better characterization of the Zn-ZnO(Nw)-rGO electrode cyclic stability, in Figure 6b is presented the calculated capacitance (Cp) reported to cycle number based on equation 1. It was demonstrated that the Zn-ZnO(Nw)-rGO electrode exhibits nonlinear capacitance behavior. After 60 cycles, the electrode capacitance decreases by 5.16%; instead, after 250 cycles, the electrode shows a retention rate of 107.36% of its initial capacitance. This indicates that over 250 cycles, the Zn-ZnO(Nw)-rGO electrode actually gains a 7.36% increase in its capacitance value, with a maximum retention rate of 111.28% obtained after 120 cycles.

**Figure 7:**
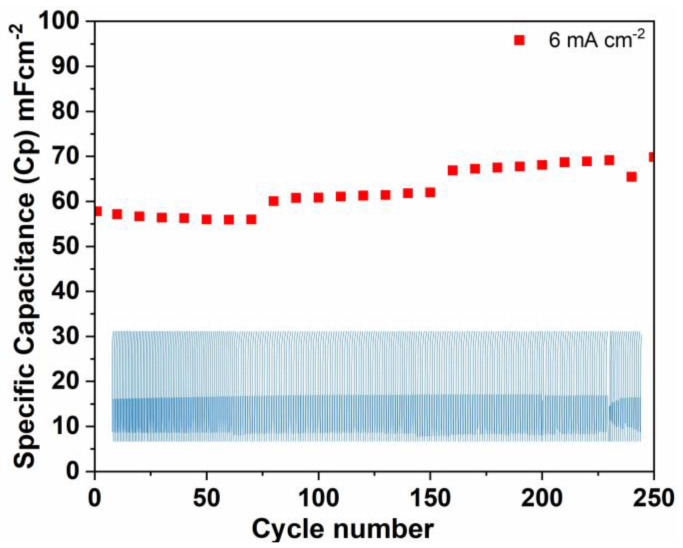
Specific capacitance at 250 cycles of the Zn-ZnO(Nw)-rGO electrode at a current density of 6 mA cm^−2^; inset GCD plot.

The cycling stability of the Zn-ZnO(Nw)-rGO hybrid electrode at a current density of 6 mA cm^−2^, evaluated over 250 cycles using GCD analysis, was presented in Figure 7. The electrode exhibited a relatively long cycle life at 6 mA cm^−2^, confirming its electrochemical stability. Interestingly, the Zn-ZnO(Nw)-rGO electrode exhibited a gradual increase in capacitance over the course of 250 cycles, with a noticeable pattern emerging around the 80th cycle. Furthermore, the electrode demonstrated a retention rate of 103.92% over the initial 80 cycles, and this value further increased to 115.69% after 160 cycles. The maximum retention rate was achieved after 250 cycles, reaching a value of 120.86%. These findings suggest that over the 250 cycles of GCD analysis, the Zn-ZnO(Nw)-rGO electrode presented a significant increase of 20.86% in its capacitance value. Moreover, these results are in accordance with the capacity increase observed in the CV analysis performed over the cycling experiments. The increase in capacitance noticed after cycling can probably be attributed to the in situ reduction of rGO during negative current cycling as well as the complete activation of the transition metal oxide (ZnO nanowires) within the Zn-ZnO(Nw)-rGO hybrid electrode [50,51]. This reduction or activation process, which involves the repeated intercalation and de-intercalation of electrolyte ions and the gradual insertion of the electrolyte into the bulk structure of ZnO (Nw), respectively, can lead to the creation of additional electrochemical active sites. The increase in active sites contributes to the increase in observed capacitance during the cycling process.

Table 1 summarizes the specific capacitance studies reported for the different morphologies of the graphene oxide composite supercapacitor.

**Figure 8:**
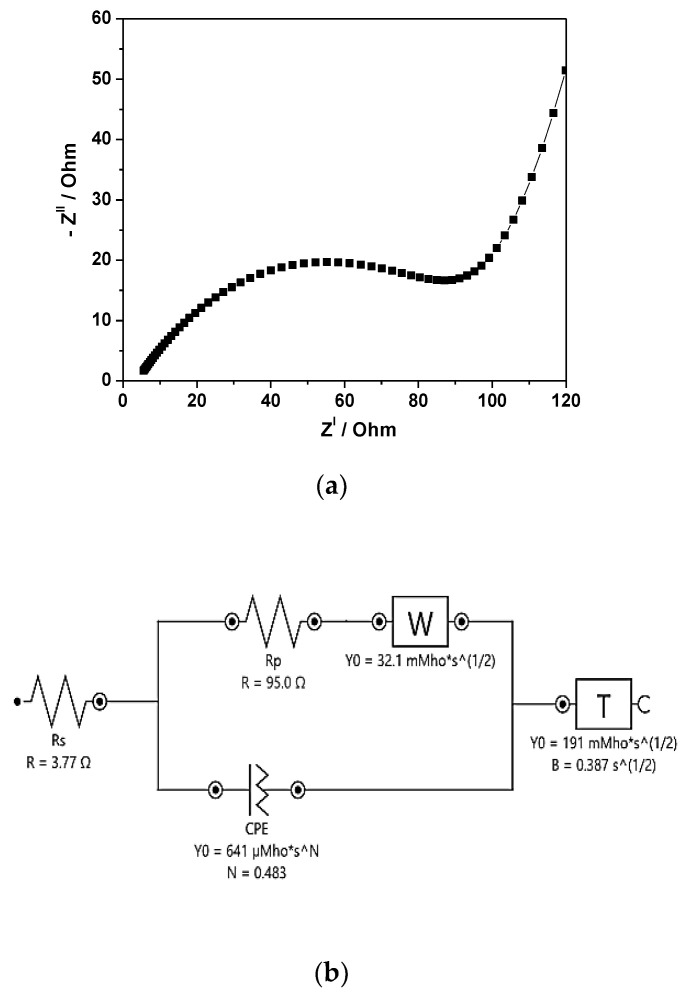
Nyquist plot (**a**) and the equivalent circuit fitting (**b**) of the Zn-ZnO(Nw)-rGO hybrid electrode.

Electrochemical impedance spectroscopy (EIS) over a frequency range of 0.1 Hz to 100.000 Hz with an amplitude of 0.01 V to gain insight into the conductivity, mechanistic analysis of interfacial processes and structure, as well as charge transport in the material/electrolyte interface, was recorded for the Zn-ZnO(Nw)-rGO hybrid electrode (Figure 8a). Figure 8b displays the equivalent circuit used for curve fitting to extract the electrochemical data from the tested hybrid electrode. The ohmic resistance of the electrode-electrolyte interface (*Rs*) on the Zn-ZnO(Nw)-rGO electrode was determined by the high-frequency intercept of the EIS plots, and the real axis was about 3.77 Ω [38]. The charge transfer resistance between the Zn-ZnO(Nw)-rGO hybrid electrode and the electrolyte (*R_P_*) was determined to be about 95 Ω from the diameter of the semicircle in Figure 8a [45,59].

The charge transfer resistance in EIS corresponds to the electrochemical activity of the active material in the system, including the impact of both redox and non-redox reactions. The slightly high value of charge transfer resistance on the negative electrode is probably due to the poor conductivity of zinc oxide [59]. The straight line observed in the low-frequency range is attributed to the Warburg resistance, caused by the frequency dependence of the ion diffusion transport from the electrolyte to the electrode surface. In addition, the slope of the electrode shows good capacitive behavior as evidenced by an inclination in the range of 45 degrees with a decrease in the frequency of the slop of the straight line [60].

## 4. Conclusions

Zn-ZnO(Nw)-rGO hybrid electrodes for supercapacitors by a one-step microwave-assisted hydrothermal deposition method of rGO directly in situ on Zn-ZnO(Nw) supports were successfully assessed within this work. According to XRD and Raman analyses, graphene oxide was reduced under microwave hydrothermal treatment directly in situ, which is accompanied by an increase in the width of the ZnO nanowires from the Zn-ZnO(Nw)-rGO hybrid electrode. The morphological result indicates a good adhesion of the reduced graphene oxide layer onto the Zn-ZnO(Nw) support and confirms the presence of ZnO nanowires on the Zn support. Moreover, in the microwave hydrothermal process, due to the synthesis conditions (temperature and pressure) in the autoclave, the exfoliation of graphene oxide into thinner graphene sheets takes place, which facilitates its deposition on the Zn-ZnO(Nw) support. In addition, from the SEM measurements, it was found that the length of the ZnO nanowires does not show significant changes before and after the hydrothermal treatment, with the main difference being observed only in the width of the nanowires. The electrochemical CV analysis reveals that the Zn-ZnO(Nw)-rGO hybrid structures work as negative electrodes and exhibit a non-ideal rectangle-like shape, suggesting that the as-synthesized structure behaves as a pseudo-capacitor. Furthermore, the maximum capacitance was calculated to be 395.79 mF cm^−2^ at a scan rate of 5 mV s^−1^.The GCD analysis indicates that the maximum specific capacitance of 145.59 mF cm^−2^ was achieved at a low power density of 2 mA cm^−2^.To evaluate the cycle life of the Zn-ZnO(Nw)-rGO electrode, a total of 250 cycles of CV and GCD analysis were performed. Within these cycles, the Zn-ZnO(Nw)-rGO electrode demonstrated non-linear capacitance behavior. The analysis of CV cycles revealed a retention rate of 107.36%, indicating that the electrode actually gains a 7.36% increase in its capacitance value from its initial capacitance after 250 cycles. Similarly, the GCD cycle analysis showed that the maximum retention rate of 120.86% was achieved after 250 cycles, indicating an even higher capacitance retention for the electrode. Furthermore, the EIS analysis demonstrates that the negative electrode exhibits favorable capacitive behavior, as evidenced by a 45-degree inclination with a decreasing frequency. Finally, the facile one-step microwave-assisted hydrothermal method, along with the morpho-structural and electrochemical properties of the Zn-ZnO(Nw)-rGO electrodes, can be considered a favorable electrode material for supercapacitor applications.

## Figures and Tables

**Table 1:** Different structures of the graphene oxide composite for supercapacitors.

Materials	Structure	Specific Capacitance (mF cm^−2^)	Scan Rate (mV s^−1^)	Specific Capacitance (mF cm^−2^)	Current Density	References
NiO/MoS_2_/rGO	Electrode	7.38	25	-	-	[52]
PPy-GO/CNT	Electrode	143.6	10	99	1.0 mA cm^−2^	[43]
rGO-CS	Electrode	25.39	2	10.61	0.5 mA cm^−2^	[53]
PEDOT/rGO-CS	Electrode	1073.67	2	584	0.5 mA cm^−2^	[53]
Sheet like ZnCO_2_O_4_	Electrode	-	-	16.13	10 µA cm^−2^	[54]
ZnO/rGO	Nano composite	-	-	0.022	1 mA cm^−2^	[55]
CFG	Electrode	-	-	1160	1 A g^−1^	[56]
Cu(OH)_2_/ graphene	Composite	-	-	317	1 mA cm^−2^	[57]
rGO-SnO_2_ SCs	Composite	-	-	37.17	0.25 mA cm^−2^	[58]
Ti_3_C_2_Tx	Electrode	1399.0	1	-	-	[51]
Zn-ZnO(Nw)-rGO	Electrode	395.79	5	145.59	2 mA cm^−2^	This work

## Data Availability

Not applicable.
